# Small left ventricular volume in HFpEF: from phenotype to clinical implications

**DOI:** 10.1093/eschf/xvag080

**Published:** 2026-03-13

**Authors:** Julio Núñez, Jan Verwerft, Gema Miñana, Rafael de la Espriella, Frederik H Verbrugge

**Affiliations:** Cardiology Department, Hospital Clínico Universitario, INCLIVA, Avenida Blasco Ibáñez 17, Valencia CP 46010, Spain; Department of Medicine, Universitat de Valencia, Avenida Blasco Ibáñez 17, Valencia CP 46010, Spain; CIBER Cardiovascular, Monforte de Lemos, 3-5, Pabellón 11, Planta 0, Madrid 28029, Spain; Department of Cardiology and Jessa & Science, Jessa Hospital, Hasselt, Belgium; Faculty of Medicine and Life Sciences/LCRC, UHasselt, Diepenbeek, Belgium; Cardiology Department, Hospital Clínico Universitario, INCLIVA, Avenida Blasco Ibáñez 17, Valencia CP 46010, Spain; Department of Medicine, Universitat de Valencia, Avenida Blasco Ibáñez 17, Valencia CP 46010, Spain; CIBER Cardiovascular, Monforte de Lemos, 3-5, Pabellón 11, Planta 0, Madrid 28029, Spain; Cardiology Department, Hospital Clínico Universitario, INCLIVA, Avenida Blasco Ibáñez 17, Valencia CP 46010, Spain; Department of Medicine, Universitat de Valencia, Avenida Blasco Ibáñez 17, Valencia CP 46010, Spain; CIBER Cardiovascular, Monforte de Lemos, 3-5, Pabellón 11, Planta 0, Madrid 28029, Spain; Centre for Cardiovascular Diseases, University Hospital Brussels, Jette, Belgium; Faculty of Medicine and Pharmacy, Vrije Universiteit Brussel, Brussels, Belgium

**Keywords:** HFpEF, Small left ventricle, Low preload reserve

## A critique of the prevailing paradigm in HFpEF

The conventional model of heart failure with preserved ejection fraction (HFpEF) attributes its main pathogenesis to cardio-renal-metabolic interactions that drive myocardial inflammation, fibrosis, concentric left ventricular (LV) hypertrophy, and increased diastolic stiffness, while relatively preserving systolic function. This prevailing paradigm assumes a uniform underlying mechanism across most HFpEF cases and relies on left ventricular ejection fraction (LVEF) as a categorical variable, thereby overlooking its continuous nature and the presence of distinct phenotypes within the preserved LVEF range. Several clinical and pathophysiological observations challenge this paradigm. First, diastolic dysfunction and LV hypertrophy alone do not fully explain the severity of symptoms or outcomes in many patients.^1^ Second, epidemiological data indicate that the risk of adverse events remains high as LVEF exceeds 60%–65%, contradicting the assumption that higher EF is invariably protective.^2^ Third, emerging evidence indicates distinct haemodynamic patterns between HF with normal and supranormal LVEF. In a cohort study, Rosch *et al*. reported that patients with LVEF 50%–60% displayed eccentric remodelling, myocardial fibrosis, and preserved stroke volume reserve, whereas those with LVEF >60%, predominantly older hypertensive women, had smaller LV volumes, increased ventricular stiffness, and impaired stroke volume augmentation.^3^ Consistent with this concept, Popovic *et al*. recently provided detailed invasive and imaging evidence in a cohort of 621 patients with HF, showing that individuals with LVEF ≥65% manifest a distinct ‘cardiac contracture’ phenotype characterized by ventricular stiffening, small cavity dimensions, and limited cardiac output reserve.^4^ Forth, a consistent lack of clinical benefit from neurohormonal antagonists, including renin-angiotensin system inhibitors and beta-blockers has been found in patients with HF and LVEF ≥60%–65% across multiple randomized trials and registries.^5^ These discrepancies suggest that the prevailing HFpEF framework may fail to capture the full spectrum of phenotypic and mechanistic heterogeneity, particularly in patients at the upper end of the LVEF spectrum.

## Reframing HFpEF: the small LV engine as a novel pathophysiological framework

**Figure 1:**
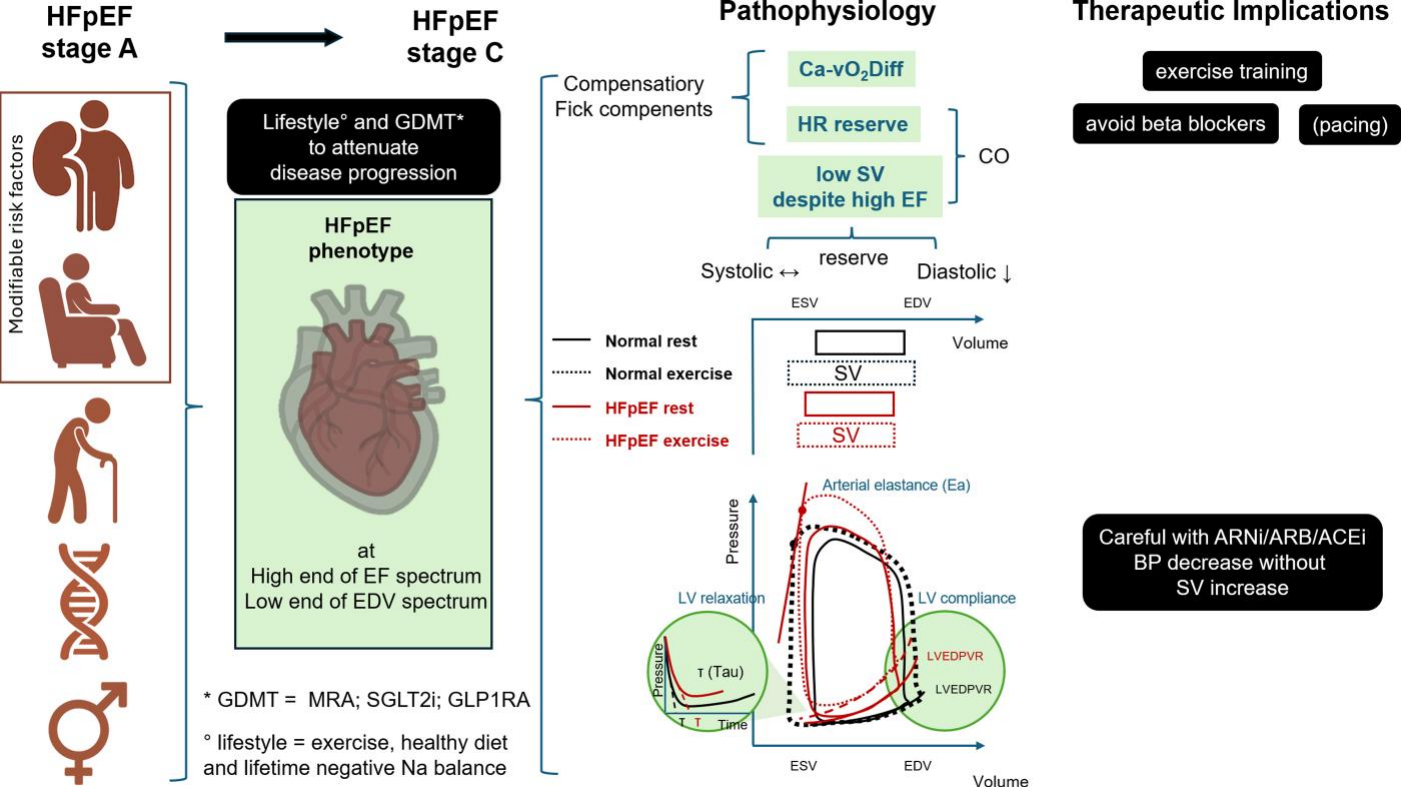
Pathophysiological framework and therapeutic implications of the small–left ventricle HFpEF phenotype. Schematic representation of the progression from HFpEF stage A to stage C, highlighting the central role of reduced left ventricular (LV) dimensions, impaired preload reserve (PR), and a leftward/upward shift of the LV end-diastolic pressure–volume relationship (LV-EDPVR), leading to limited stroke volume (SV) augmentation. The figure also summarizes potential therapeutic implications, emphasizing lifestyle interventions (exercise training, healthy diet, and long-term negative sodium balance) and pharmacological treatment, including mineralocorticoid receptor antagonists (MRA), sodium–glucose cotransporter-2 inhibitors (SGLT2i), and glucagon-like peptide-1 receptor agonists (GLP-1 RA) to attenuate disease progression. HFpEF: heart failure with preserved ejection fraction

We advance a novel, testable hypothesis that a small LV represents a central and primary determinant of a distinct HFpEF phenotype. From fundamental physiological principles, reduced LV dimensions confer an intrinsically lower preload reserve and a limited capacity to augment stroke volume during exercise or stress, accompanied by a leftward shift of the LV end-diastolic pressure–volume relationship, indicative of reduced diastolic capacitance (*Figure 1*). Nature provides striking examples of organisms with small LVs in which cardiac output is sustained primarily through chronotropic reserve.^6^ Thereby, ‘supranormal’ ejection fraction and tachycardia may be interpreted as compensatory adaptations, rather than primary pathophysiologic drivers, aiming to preserve forward output in the context of reduced preload reserve. Within this framework, LV hypertrophy and conventional indices of diastolic dysfunction would primarily exacerbate the underlying preload limitation, rather than constitute the principal determinant of disease severity in patients with small LVs. This stands in contrast to the athlete’s heart, where sustained high cardiac output demands promote physiological chamber enlargement and enhanced diastolic reserve.

Multiple observations in HFpEF support this conceptual shift. First, a strong inverse relationship between LV size and supranormal ejection fraction has been consistently reported.^2–4,7^ Second, the age-related reduction in LV cavity dimensions and increase in chamber stiffness may partially explain the greater prevalence of HF with supranormal LVEF among older individuals.^8^ Third, because women typically exhibit smaller LV cavities, this framework provides a mechanistic basis for the sex-specific pattern characterized by higher LVEF and resting heart rate, yet lower stroke volume and reduced cardiac output reserve in women.^2,9^ Thus, it offers a coherent explanation for sex-related differences in epidemiology, prognosis, and therapeutic response in HFpEF.^9^ Fourth, the inverse association between indexed LV volumes and body mass index suggests a potential structural pathway linking obesity to the increased susceptibility to HFpEF in this population. The respective contributions of genetic predisposition versus acquired remodelling in determining LV size remain to be elucidated.

## Clinical evidence

Recent studies have highlighted the crucial role of a smaller LV in predicting reduced functional capacity and a higher risk of adverse outcomes in different clinical scenarios. In an elegant cardiac magnetic resonance (CMR) study by Foulkes *et al*. in 185 healthy women, peak VO_2_ correlated rather strongly with LV and right ventricular end-diastolic volumes (*R*^2^ = 0.58–0.63; *P* < .001) but weakly with LV systolic/diastolic function.^10^ In this study, smaller LVEDV was associated with reduced cardiac reserve, limiting stroke volume and limiting augmentation of output during exercise.^10^ More recently, Rowe *et al*. reported in a multicentre study that included 2876 participants, including athletes, healthy controls, and patients with dyspnoea or HFpEF, indexed LV end-diastolic volumes (iLVEDV) showed the strongest association with peak VO_2_ (*R*^2^ = 0.45, *P* < .001) and remained the strongest independent predictor after adjusting for confounders.^11^ Importantly, although LVEDV is a principal determinant of peak cardiac output, peak VO_2_ is determined by the interaction of cardiac output and the arteriovenous oxygen difference.

Regarding clinical outcomes, in a pooled analysis of 6990 participants from two large population-based cohorts, Shah *et al*. showed that supranormal LVEF was linked to a higher risk of adverse cardiovascular outcomes, with the excess risk concentrated among individuals with low stroke volume reflecting smaller left ventricular dimensions and diminished preload reserve.^2^ Rowe *et al*. examined a large multicentre echocardiographic cohort of 366 484 individuals and revealed that smaller LV volumes, particularly in females and patients with high-normal LVEF (≥60%), were associated with increased 5-year all-cause and cardiovascular mortality.^12^ Longitudinal data also suggest that progressive reductions in LV size over time, even within the normal range, are associated with higher risk of mortality and CV hospitalizations, underscoring the prognostic relevance of declining LV dimensions with ageing.^8^ More specifically, a recent study that comprised 5471 individuals without a history of HF, smaller iLVEDV, assessed by CMR, was associated with a higher risk of incident HF in those with CMR-LVEF >60%.^7^ Dedicated prospective investigations are required to explore the association between smaller LV size and the risk of incident HF and long-term adverse outcomes in symptomatic HFpEF.

## Potential clinical implications

While current diagnostic consensus statements for HFpEF recognize increased relative wall thickness as a structural criterion, they do not explicitly incorporate reduced LV chamber dimensions into the diagnostic workflow. The role of natriuretic peptides for diagnosis, risk stratification, and monitoring also requires dedicated evaluation in this subgroup of patients. From a therapeutic perspective, subgroup analyses of recent HFpEF trials have consistently shown that renin–angiotensin antagonists provide limited or no benefit in patients with higher LVEF.^5^ Heart rate reduction with beta blockers has been associated with adverse clinical outcomes at the upper ranges of LVEF. For instance, in a large registry-based analysis including 435 897 patients with HF and LVEF ≥40%, Arnold *et al*. reported that beta blocker use was linked to higher mortality and to the composite of mortality/HF hospitalizations in those with higher LVEF.^13^ Similarly, a prespecified substudy of the PRESERVE-HR randomized trial found that in HFpEF patients with chronotropic incompetence, smaller LV volumes identified those who achieved the greatest short-term improvement in maximal functional capacity after beta blocker withdrawal.^14^ Also, strategies aimed at increasing heart rate, either through rate-adaptive pacing or pharmacologic interventions, have shown potential benefits in this phenotype. For example, in a secondary analysis of the myPACE trial, which randomized patients with stage B/C HFpEF and pacemakers to individualized accelerated pacing versus usual care, improvements in quality of life, natriuretic peptides, and device-detected activity were most pronounced in patients with smaller iLVEDV and higher LVEF, suggesting preferential benefit in this distinct HFpEF phenotype.^15^

Finally, exercise training may represent a highly relevant non-pharmacological strategy in this context. A post-hoc analysis of the randomized TRAINING-HR trial in older symptomatic HFpEF patients with chronotropic incompetence (*n* = 80), most of whom displayed small LV volumes at baseline, showed that 12-week supervised aerobic ± resistance training (vs exercise advice) increased LVEDVi by ∼3–8 ml/m^2^ (≈10%–20%), with LV expansion independently correlating with peak VO_2_ gains.^16^

## Proposed directions for research

To validate the small LV hypothesis, prospective studies are needed to (i) comprehensively characterize resting and exercise haemodynamics in individuals with smaller LV dimensions; (ii) delineate the trajectory of cardiac chamber size across the lifespan, including the influence of genetic and environmental determinants; (iii) establish diagnostic thresholds to define pathologically small LVs; (iv) evaluate the diagnostic and prognostic performance of conventional biomarkers, such as natriuretic peptides, in individuals with smaller LVs at risk or with symptomatic HF; (v) investigate the contributions of myocardial stiffness, pericardial constraint, and altered protein synthesis pathways to this phenotype; (vi) assess the efficacy and safety of different decongestion strategies and guideline-directed medical therapies in this specific subset; and (vii) explore interventions aimed at enhancing LV compliance, remodelling, and chronotropic reserve, including tailored exercise training (NCT04745013), pericardial interventions, and heart rate augmentation (NCT06099891) in dedicated prospective studies.

## Declarations

### Data Availability

No data were generated or analysed for this manuscript.
